# Validation and Psychometric Properties of the Portuguese Version of the Coronavirus Anxiety Scale (CAS) and Fear of COVID-19 Scale (FCV-19S) and Associations with Travel, Tourism and Hospitality

**DOI:** 10.3390/ijerph18020427

**Published:** 2021-01-07

**Authors:** José Magano, Diogo Guedes Vidal, Hélder Fernando Pedrosa e Sousa, Maria Alzira Pimenta Dinis, Ângela Leite

**Affiliations:** 1 ISCET—Higher Institute of Business Sciences and Tourism, Rua de Cedofeita, 285, 4050-180 Porto, Portugal; 2 Research Center in Business and Economics (CICEE), Universidade Autónoma de Lisboa, Rua Sta. Marta 47, 5.° Andar, 1150-293 Lisboa, Portugal; 3 UFP Energy, Environment and Health Research Unit (FP-ENAS), University Fernando Pessoa (UFP), Praça 9 de Abril 349, 4249-004 Porto, Portugal; diogovidal@ufp.edu.pt; 4 Department of Mathematics (DM.UTAD), University of Trás-os-Montes and Alto Douro (UTAD), Quinta de Prados, 5001-801 Vila Real, Portugal; hfps@utad.pt; 5 School of Human and Social Sciences (ECHS), University of Trás-os-Montes and Alto Douro (UTAD), Quinta de Prados, 5001-801 Vila Real, Portugal; angelal@utad.pt

**Keywords:** Coronavirus Anxiety Scale (CAS), Fear of COVID-19 Scale (FCV-19S), travel, tourism, hospitality

## Abstract

The aim of this study is to determine the anxiety and fear related to coronavirus disease 2019 (COVID-19) and their associations with travel, tourism and hospitality, in the Portuguese population. The Coronavirus Anxiety Scale (CAS) and Fear of COVID-19 Scale (FCV-19S) were validated for the Portuguese population and correlations with issues related to travel, tourism and hospitality were established. CAS and FCV-19S presented a good adjustment model and solid reliability and validity. Correlations between CAS and FCV-19S and the perception of the impact of COVID-19 in travel, tourism and hospitality were found. Participants considered that COVID-19 mainly affected their holidays and leisure time. However, the strongest correlation established was between total FCV-19S and emotional fear FCV-19S and the fear of attending hotel facilities. The Portuguese versions of CAS and FCV-19S are reliable psychological tools to assess anxiety and fear in relation to COVID-19 for the general population. The use of hotel facilities is the most threatening issue related to travel, tourism and hospitality. The results suggest that hotels should invest in hygiene and safety measures that allow users to regain confidence in hotel equipment.

## 1. Introduction

The severe acute respiratory syndrome coronavirus 2 (SARS-CoV-2) is the virus causing coronavirus disease 2019 (COVID-19), the disease that was first discovered in 2019 in China [1]. As of December 28th, 2020, there were 80,879,693 people infected with this virus around the world, and 1,766,787 died from it. In Portugal, 394,573 people were infected, and 6619 died [2]. Thus, the COVID-19 pandemic is a massive global health crisis [3]. The most common symptoms at the onset of COVID-19 are fever, cough and fatigue [4], although other symptoms include headache, hemoptysis, diarrhea, dyspnea and lymphopenia [5]. Being male, elderly and having comorbidities have been significantly associated with the risk of death among COVID-19 patients [6].

As a result of the emergence of the COVID-19 outbreak, a socio-economic crisis and profound psychological distress occurred worldwide [7]. People who became infected with the virus developed psychological disorders associated with the general situation (isolation, loss of income, loneliness) [8] and their particular situation (fear, uncertainty, anxiety, depression and post-traumatic stress) [9]. People who did not become infected saw their family and friends get sick, some of whom even died, triggering feelings of helplessness, anxiety and fear [10,11].

Anxiety and fear about COVID-19 had other implications for people’s daily lives, namely, concerning travel (for work, leisure or holidays) [12,13] and hospitality [14,15]. According to Zheng, Luo and Ritchie [9], threat severity and susceptibility may cause “travel fear”, leading to protection motivation and protective travel behaviors, even after the pandemic outbreak.

Some authors felt the need to develop tools to assess anxiety and fear in the face of COVID-19. Lee [16] conceived the Coronavirus Anxiety Scale (CAS), a brief mental health screener that can be used to identify cases of dysfunctional anxiety related to COVID-19. This scale has already been adapted to the Bangla [17], Korean [18], Turkish [19] and Polish [20] languages. 

Additionally, Ahorsu and colleagues [10] developed the Fear of COVID-19 Scale (FCV-19S) to identify and early intervene, psychologically, in people with high values of fear of COVID-19. This scale has been validated in the Bangla [21], Greek [22], Arabic [23], Malay [24], Italian [25], Hebrew [26] and Spanish [27] languages, Spanish language in Peru [28], Spanish language in Argentina [29], Portuguese language in Brazil [30], Japanese [31] and Chinese [32] languages and in Eastern Europe [33] and India [34].

This study aims to determine the anxiety and fear related to COVID 19 and its associations with travel, tourism and hospitality, in the Portuguese population. To this end, instruments have been validated for the Portuguese population to assess anxiety and fear in relation to COVID-19. Then, the validated tools have been associated with questions related to travel, tour-ism and hospitality. It has been hypothesized that a good model of adjustment for CAS and FCV-19S would be found for the Portuguese population *(H1)* and it has also been hypothesized that high levels of anxiety and fear related to COVID-19 would be positively associated with a greater perception of the pandemic’s impact on travel, tourism and hospitality *(H2).*

## 2. Materials and Methods

### 2.1. Procedures

Permission was received from the original authors [10,16] to validate the instruments in a Portuguese population. It was then translated from English to Portuguese using the back-translation technique [35] (Appendix A, Table A1 and Table A2; Appendix B, Table A3 and Table A4). After the protocol was conceived, including the sociodemographic questionnaire, issues related to travel, tourism and hospitality, CAS and FCV-19S, it was submitted to the ethics committee of the University of Trás-os-Montes and Alto Douro (UTAD), having been approved in 1 September 2020. It was released to the general population through a social network page about the study, with data being collected between 1 October and 15 November 2020. Convenience and snowball samplings were used. Consequently, the sample is not representative of the Portuguese population. All procedures performed in this study were in accordance with the ethical standards of the institutional research committee and with the 1964 Helsinki Declaration and its later amendments. Informed consent, in which the participants were familiarized with the aims of the study and in which the confidentiality and anonymity of the data were guaranteed, as well as the strategy to destroy data after being used, preceded the investigation protocol and the participants only accessed it after giving their consent.

### 2.2. Instruments

#### 2.2.1. Sociodemographic Questionnaire

This questionnaire included questions related to gender (man vs. woman), age (numerical), education (no university studies vs. university studies) and employment status (inactive—unemployed, sick, retired, on medical leave—vs. active—students, employees, housewives).

#### 2.2.2. Questions Related to Travel, Tourism and Hospitality

Seven questions were designed to assess the participants’ perceptions of the impact of COVID-19 on travel, tourism and hospitality in times of the COVID-19 pandemic. The instruction (“On a scale of 0 to 100, please indicate how much the pandemic situation caused by COVID-19 has…”) proceeded with the items: 1—… “changed your leisure activities”; 2—… “changed your vacations”; 3—… “prevented you from settling in a hotel”; 4—… “prevented you from traveling by plane”; 5—… “prevented you from traveling by train”; 6—… “prevented you from traveling by car”; and 7—… “made you feel fear of attending hotel facilities”.

#### 2.2.3. Coronavirus Anxiety Scale (CAS) 

The CAS was developed with the purpose of filling a void in the mental health response to COVID-19 [16]. According to Lee [16], a brief mental health screener that could identify probable cases of dysfunctional anxiety and symptom severity associated with the coronavirus was needed. This is a five-item scale that assesses distinct physiological reactions of anxiety related to COVID-19, highly reliable as a cluster (*α* = 0.93) [16].

#### 2.2.4. Fear of COVID-19 Scale (FCV-19S) 

According to Ahorsu and colleagues [10], FCV-19S was developed to complement the clinical efforts in preventing the spread of and treating COVID-19 cases. This is a seven-item scale, with robust psychometric properties (composite reliability (*CR*)—0.88; average variance extracted (*AVE*)—0.5; internal consistency [Cronbach’s α]—0.82; standard error of measurement (*SEM*)—1.89; item separation reliability (*SR*) from Rasch—0.99; item separation index (*SI*) from Rasch—11.45; person separation reliability (*PSR*) from Rasch—0.77; and person separation index (*PSI*) from Rasch—2.82), being reliable and valid in assessing fear of COVID-19 among the general population and useful in alleviating COVID-19 fears among individuals.

### 2.3. Analytical Approach

An internal replicability approach was employed by subjecting one half of the study’s data to an exploratory factor analysis (EFA) and the other half to confirmatory factor analysis (CFA) to address the sampling error influences. The EFA was used to identify representative symptoms of coronavirus anxiety, while the CFA was used to test the replicability of the EFA results. The second half of the study’s data was also used to perform a series of multiple group CFAs to determine if the construct of coronavirus anxiety presents differences across demographic groups. Pearson correlations between questions related to travel, tourism and hospitality and anxiety and fear towards COVID-19 were performed, as well as Spearman correlations between anxiety and fear and sociodemographic variables. Test–retest reliability was calculated using Pearson correlations to assess the CAS and FCV-19S constructs’ stability and precision across time. According to the guidelines suggested by Vaz et al. [36], if the *p*-value is less than 0.05, and the Pearson correlation coefficient is above 0.7, then researchers have evidence of test–retest reliability. Statistical analyses were calculated using Statistical Program for Social Sciences SPSS version 27.0 (IBM Corp., Armonk, NY, USA), and CFA was run using AMOS version 27.0 (IBM Corp., Armonk, NY, USA).

## 3. Results

### 3.1. Sample

Two independent samples with the same number of participants were used. As a whole, the sample consisted of 1122 participants, of whom 725 (64.6%) are women, with a mean age of 31.91 years of age (*SD* = 13.76), with 495 (44.1%) having university studies and the remaining (*n* = 627; 55.9%) without.

Concerning professional status, 932 (83.1%) are active and the remaining are inactive. The EFA sample (*n* = 561) was not different from the CFA sample (*n* = 561) in relation to sociodemographic issues (chi-square tests and Student’s *t* tests), except for age [*t*(1111, 613) = 2.22; *p* = 0.027; *d* = 0.13], being that the EFA sample was slightly older (*M* = 32.82 years old; *SD* = 14.32) than the CFA sample (*M* = 31.00 years old; *SD* = 13.12). 

#### 3.1.1. Exploratory Factor Analysis (EFA) Results: Coronavirus Anxiety Scale (CAS)

Data screening results suggested that the five items were suitable for EFA [37]: no issues relating to sample size, missing data, nonnormality, multicollinearity or singularity. The correlation matrices were factorable [Bartlett’s test of sphericity = *p* < 0.001; Kaiser Meyer–Olkin (KMO) test = 0.85)], being that Goretzko et al. [38] suggested the following values, in EFA: factor loading > 0.5; KMO ≥ 0.5; Bartlett’s test of sphericity to assess the statistical significance < 0.05; percentage of variance in extraction sums of squared loadings > 50%.

The five items of the CAS were subjected to an EFA with varimax rotation. The maximum likelihood factor analysis with a cut-off point of 0.40 and the Kaiser’s criterion of eigenvalues greater than 1 [39] yielded a one-factor solution as the best fit for the data, accounting for 67.64% of total variance explained. The five items meet the criteria for psychometrically sound items (Table 1). Structure coefficients ranged from 0.73 to 0.89, and communality coefficients ranged from 0.53 to 0.79. Correlations between items ranged from 0.45 to 0.73. These items were reliable as a single dimension (*α* = 0.85). If any item was deleted, alpha’s value decreased.

The test–retest agreement was analysed item by item between the first and second (*n* = 31) evaluations. The correlations between the first and the second moments were all over *r* = 0.70 and the significance was always above *p* = 0.50.

#### 3.1.2. Confirmatory Factor Analysis (CFA) Results: Coronavirus Anxiety Scale (CAS)

**Figure 1:**
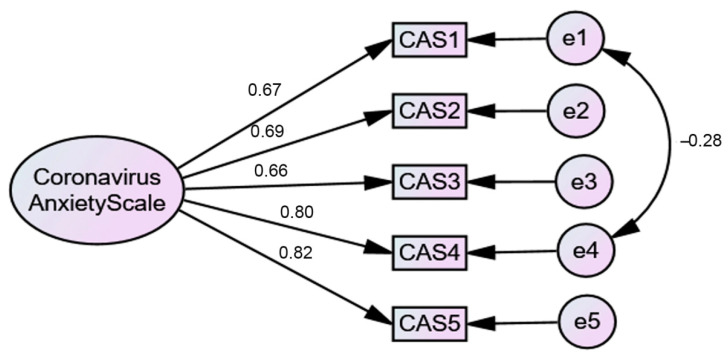
One-factor CFA model.

To test the model found in EFA, a CFA was performed. The results supported the EFA findings (Figure 1). A one-factor model was found [*χ*^2^(4) = 7.67, *p* = 0.11] with an excellent fit for all of the indices [*χ*^2^/*df* ratio = 1.92; Comparative Fit Index (*CFI*) = 1.00; Tucker Lewis Index (*TLI*) = 0.99; Standardized Root-Mean-Square Residual (*SRMR*) = 0.02; Root-Mean-Square Error of Approximation (*RMSEA*) = 0.04 (0.00, 0.05; 90% Confidence Interval (*CI*)); *p*-value of Close Fit (*PCLOSE*) = 0.58]. However, to achieve this model, a correlation between two items’ errors (items 1 and 4) was established. To test if the coronavirus anxiety construct was measured the same way across genders (women vs. men), multigroup CFAs were performed. The results demonstrated gender differences, which were evidenced by the model fit [*χ*^2^(8) = 21.90, *p* = 0.05] [*χ*^2^/*df* ratio = 2.74; *CFI* = 0.99; *TLI* = 0.98; *SRMR* = 0.04; *RMSEA* = 0.06 (0.00, 0.06; 90% *CI*); *PCLOSE* = 0.33] and a significant increase in the *χ*^2^ value [Δ*χ*^2^ (4) = 27.79, *p* < 0.001] between the models. Women (*M* = 1.32; *SD* = 0.50) presented higher levels of anxiety related to COVID-19 than men (*M* = 1.14; *SD* = 0.31).

#### 3.1.3. Exploratory Factor Analysis (EFA) Results: Fear of COVID-19 Scale (FCV-19S)

Data screening results suggested that the seven items were suitable for EFA [37]: again, no issues relating to sample size, missing data, nonnormality, multicollinearity or singularity. The correlation matrices were also factorable [Bartlett’s test of sphericity = *p* < 0.001; Kaiser Meyer–Olkin (KMO) test = 0.85)].

The seven items of FCV-19S were subjected to an EFA with varimax rotation. The maximum likelihood factor analysis with a cut-off point of 0.40 and the Kaiser’s criterion of eigenvalues greater than 1 [39] yielded a two-factor solution as the best fit for the data, accounting for 70.72% of total variance explained. The seven items meet the criteria for psychometrically sound items (Table 2). Structure coefficients ranged from 0.71 to 0.86, and communality coefficients ranged from 0.57 to 0.79. Correlations between items ranged from 0.37 to 0.72. These items were reliable as a single dimension (*α* = 0.88) (if any item was deleted, alpha’s value decreased) and as two-factor dimensions (first one *α* = 0.83; second one *α* = 0.82).

The test–retest agreement was analysed item by item between the first and second (*N* = 31) evaluations. The correlations between the first and the second moments were all over *r* = 0.70 and the significance was always above *p* = 0.50. 

#### 3.1.4. Confirmatory Factor Analysis (CFA) Results: Fear of COVID-19 Scale (FCV-19S)

**Figure 2:**
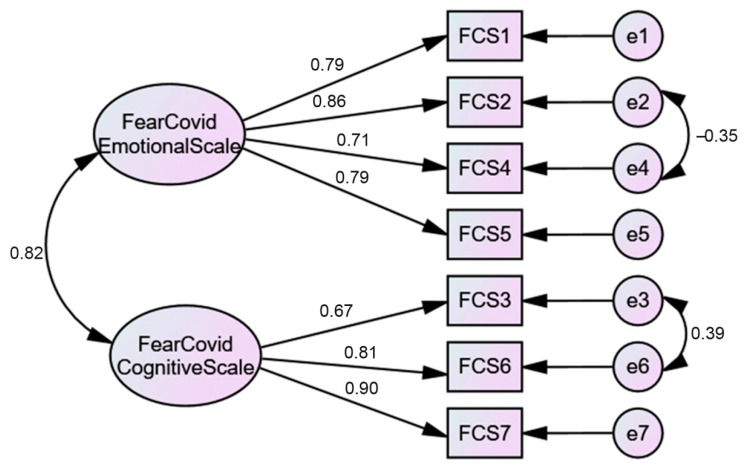
Two-factor CFA model.

To test the model found in EFA, a CFA was performed. The results supported the EFA findings (Figure 2). A two-factor model was found [*χ*^2^(11) = 39.56, *p* < 0.001] with a moderate fit for all of the indices [*χ*^2^/*df* ratio = 3.58; *CFI* = 0.99; *TLI* = 0.97; *SRMR* = 0.03; *RMSEA* = 0.07 (0.00, 0.05; 90% *CI*); *PCLOSE* = 0.09]. However, to achieve this model, a correlation between two items’ errors (items 2 and 4) of the first factor and a correlation between two items’ errors of the second factor (items 3 and 6) were established. As the items whose errors were correlated belonged to the same factor, in theory, the correlation is justified. To test if the fear of COVID-19 construct was measured the same way across genders (women vs. men), multigroup CFAs were performed. The results demonstrated gender differences, which were evidenced by the model fit [*χ*^2^(22) = 44.11, *p* = 0.03] [*χ*^2^/*df* ratio = 2.01; *CFI* = 0.99; *TLI* = 0.98; *SRMR* = 0.04; *RMSEA* = 0.04 (0.00, 0.06; 90% *CI*); *PCLOSE* = 0.74], although the increase in the *χ*^2^ value [Δ*χ*^2^ (5) = 7.93, *p* < 0.16] between the models was not significant. Women (*M* = 2.66; *SD* = 0.83) presented higher levels of fear of COVID-19 than men (*M* = 2.14; *SD* = 0.79).

#### 3.1.5. Correlations

CAS correlates positively with FCV-19S (*r* = 0.53; *p* < 0.001); with emotional fear (*r* = 0.43; *p* < 0.001); and with cognitive fear (*r* = 0.57; *p* < 0.001). CAS correlates positively with gender (*r*_s_ = 0.15; *p* < 0.001); additionally, FCV-19S correlates positively with gender (*r*_s_ = 0.26; *p* < 0.001), as well as emotional fear (*r*_s_ = 0.29; *p* < 0.001) and cognitive fear (*r*_s_ = 0.18; *p* < 0.001). No other correlations between CAS and FCV-19S with the selected sociodemographic variables were found.

Correlations between the seven questions about travel, tourism and hospitality range from *r* = 0.19 to *r* = 0.63, being all the correlations significative at the *p* < 0.001 level. Cronbach’s alpha suggests good reliability (Table 3). All questions concerning travel, tourism and hospitality correlate positively with the CAS and FCV-19S and their dimensions (Table 3). The highest correlations were found between FCV-19S and emotional fear, on the one hand, and attending hotel facilities, on the other hand. The correlations between the same dimensions and avoiding settling in a hotel follow. However, if paying attention to the means of the items related to travel, tourism and hospitality (Table 3), it turns out that the highest mean relates to the impact of COVID-19 on vacations and leisure time; the lowest mean refers to traveling by car.

#### 3.1.6. Regressions

Seeking to find the variables that explained the anxiety related to COVID-19, a multiple linear regression was performed and explained 7.2% of the anxiety variance [*F*(2, 558) = 22.57; *p* < 0.001], being that the variables gender (*β* = 0.17; *p* < 0.000) and use of hotel equipment (*β* = 0.20; *p* < 0.000) contribute significantly to the explanation of this variance (*R*^2^ = 0.07). The same was performed for fear of COVID-19 and it was found that gender (*β* = 0.24; *p* < 0.000), age (*β* = 0.07; *p* = 0.050), leisure (*β* = 0.12; *p* < 0.000) and use of hotel equipment (*β* = 0.37; *p* < 0.000) contribute significantly to the explanation (*R*^2^ = 0.26) of the fear of COVID-19 variance. Considered together, these variables explain 26% of the variance of fear of having COVID-19.

## 4. Discussion

This study aims to determine the anxiety and fear associated with COVID-19 and their associations with travel, tourism and hospitality, in the Portuguese population. The study is particularly important because there were no tools to assess these constructs for the Portuguese population. Accordingly, two instruments to evaluate anxiety and fear in relation to COVID-19 were assessed and validated. CAS [16] is a five-item scale assessing distinct physiological reactions of anxiety related to the coronavirus. FCV-19S [10] is a seven-item scale assessing fear of COVID-19 among the general population. In both instruments, a high score means more anxiety and more fear, respectively. Both CFAs of the original one-factor model for CAS and the two-factor model for FCV-19S, proposed by the authors of the original scale [10,16], showed a good fit with the most important indices, thus confirming the first stated hypothesis, *H1*. Additionally, both instruments revealed good internal consistency for the global score and the two subscales of the FCV-19S. The findings are consistent with those found for CAS in a significant number of studies [16,17,18,19,20,21,22,24,25,27,28,29,31,32,33,34].

The validated tools were subsequently associated with questions related to travel, tourism and hospitality. All questions concerning travel, tourism and hospitality correlated positively with the CAS and FCV-19S and their dimensions, confirming the second hypothesis, *H2*, and corroborating previous studies [15,40,41]. Participants reported that the items most impacted by COVID-19 were vacations and leisure time, and the item lesser impacted was traveling by car, corroborating these results in the literature concerning leisure [42] and traveling by car [43]. In fact, leisure activities and holidays have undergone profound changes. However, although care shares have lowered in frequency, people continue to use their car alone to go to work. The strongest correlation was found between FCV-19S and emotional fear, on the one hand, and attending hotel facilities, on the other hand. Interestingly, this correlation with emotional fear suggests that, regardless of the measures that the hotel industry may be willing to carry out, emotional fear overlaps, which does not mean that the hotel industry should not continue to invest in hygiene and safety measures that guarantee users to regain confidence in such equipment, aiming to overcome resistance from clients.

These results are just a glimpse of how this pandemic has affected people’s daily lives. All changes are generating anxiety and fear, especially when they are unwanted, as most of the changes that people have had to carry out in their life related to COVID-19 [43]. The change process alone causes anxiety. When the reason for the change causes fear, this overlap of anxiety and fear can be quite disturbing and can have lasting negative consequences. In fact, fear increases anxiety in healthy individuals and intensifies the symptoms of those with pre-existing psychiatric disorders.

## 5. Conclusions

In conclusion, this study provides the CAS and FCV-19S Portuguese versions as reliable and valid instruments, useful for measuring anxiety and fear related to COVID-19. The instruments showed good fit indices in the factor structure. The results also show good consistency indices for global scores and FCV-19S subscales. As far as it is known, these are the first instruments validated in a Portuguese population which evaluate anxiety and fear related to COVID-19. This study has some limitations. It is a cross-sectional design that hinders interpreting causality, and the questionnaire was self-applied. Future studies should test the fit of the instruments in a clinical sample.

## Figures and Tables

**Table 1:** CAS EFA results.

Items	Anxiety Symptom	*LD*	*h* ^2^	*M*	*SD*	*S_kw_*	*K_rt_*	*Min*	*Max*
Item 1	Dizziness	0.89	0.53	1.12	0.42	4.94	29.61	1	5
Item 2	Sleep Disturbances	0.89	0.60	1.43	0.74	2.33	6.19	1	5
Item 3	Tonic Immobility	0.82	0.67	1.31	0.62	2.54	7.14	1	5
Item 4	Appetite Loss	0.77	0.79	1.20	0.54	4.19	20.76	1	5
Item 5	Abdominal Distress	0.73	0.79	1.23	0.58	3.64	15.26	1	5
Total	CAS			6.29	2.35				

Notes: *LD* = structure coefficients; *h*^2^ = extracted communality coefficients; *M* = mean; *SD* = standard deviation; *S_kw_* = skewness; *K_rt_* = kurtosis; *Min* = minimum; *Max* = maximum; CAS = Coronavirus Anxiety Scale.

**Table 2:** FCV-19S EFA results.

*Items*	Fear Items	*LD1*	*LD2*	*h* ^2^	*M*	*SD*	*S_kw_*	*K_rt_*	*Min*	*Max*
Item 1	Fear of COVID-19	0.86		0.76	3.16	1.10	−0.23	−0.53	1	5
Item 2	Uncomfortable	0.81		0.71	3.01	1.07	−0.29	−0.56	1	5
Item 3	Clammy hands		0.82	0.70	1.68	0.77	1.30	2.04	1	5
Item 4	Fear of dying	0.71		0.57	2.65	1.18	0.20	−0.81	1	5
Item 5	News anxiety	0.71		0.67	2.77	1.11	−0.04	−0.77	1	5
Item 6	Trouble sleeping		0.86	0.79	1.79	0.88	1.22	1.33	1	5
Item 7	Tachycardia		0.77	0.75	2.03	1.08	0.98	0.23	1	5
Total	FCV-19S				17.20	5.69				
Factor 1	1, 2, 4, 5 items	Emotional fear			2.90	0.91				
Factor 2	3, 6, 7 items	Cognitive fear			1.84	0.79				

**Notes:***LD* = structure coefficients; *h*^2^ = extracted communality coefficients; *M* = mean; *SD* = standard deviation; *S_kw_* = skewness; *K_rt_* = kurtosis; *Min* = minimum; *Max* = maximum; FCV-19S = Fear of Covid-19 Scale.

**Table 3:** Mean of COVID-related impediments and its Pearson correlations with CAS and FCV-19S.

Instruction: on a Scale of 0 to 100 Please Indicate How Much the Pandemic Situation Caused by COVID-19…	*M*	*SD*	CAS	FCV-19S	Emotional Fear	Cognitive Fear
1—changed your leisure activities	71.59	22.49	0.12	0.27	0.29	0.19
2—changed your vacations	72.54	29.79	0.09	0.19	0.20	0.12
3—prevented you from settling in a hotel	49.26	38.97	0.19	0.31	0.32	0.23
4—prevented you from traveling by plane	67.30	39.49	0.10	0.19	0.21	0.11
5—prevented you from traveling by train	47.06	34.83	0.16	0.25	0.26	0.19
6—prevented you from traveling by car	14.39	22.01	0.16	0.21	0.17	0.21
7—made you feel fear of attending hotel facilities	45.22	33.94	0.23	0.42	0.44	0.29
Cronbach’s alpha = 0.80						

Notes. *M* = mean; *SD* = standard deviation; CAS = Coronavirus Anxiety Scale; FCV-19S = Fear of COVID-19 Scale; all correlations are significant at the *p* < 0.001 level.

## Data Availability

The data presented in this study are available on request from the corresponding author. The data are not publicly available due to General Data Protection Regulation (GDPR).

## Appendix A

Fear of COVID-19 Scale

### Appendix A.1. English Version

Please indicate your level of agreement with the statements:

**Table A1:** Items of the Fear of COVID-19 Scale (English version).

Answers	Strongly Disagree	Disagree	Neither Agree nor Disagree	Agree	Strongly Agree
1. I am most afraid of Corona.	□	□	□	□	□
2. It makes me uncomfortable to think about Corona.	□	□	□	□	□
3. My hands become clammy when I think about Corona.	□	□	□	□	□
4. I am afraid of losing my life because of Corona.	□	□	□	□	□
5. When watching news and stories about Corona on social media, I become nervous or anxious.	□	□	□	□	□
6. I cannot sleep because I’m worrying about getting Corona.	□	□	□	□	□
7. My heart races or palpitates when I think about getting Corona.	□	□	□	□	□

### Appendix A.2. Portuguese Version

Por favor, assinale a sua concordância ou discordância em relação às seguintes afirmações:

**Table A2:** Items of the Fear of COVID-19 Scale (Portuguese version).

Respostas	Discordo Completamente	Discordo	Não Concordo nem Discordo	Concordo	Concordo Completamente
1. Tenho muito medo do COVID-19.	□	□	□	□	□
2. Fico desconfortável quando penso no COVID-19.	□	□	□	□	□
3. As minhas mãos ficam húmidas quando penso no COVID-19.	□	□	□	□	□
4. Tenho medo de perder a vida por causa do COVID-19.	□	□	□	□	□
5. Ao assistir às notícias e histórias sobre o COVID-19 nas redes sociais, fico nervoso(a) ou ansioso(a).	□	□	□	□	□
6. Não consigo dormir porque estou preocupado com a possibilidade de ficar infetado(a) com COVID-19.	□	□	□	□	□
7. O meu coração dispara ou palpita quando penso na possibilidade de ficar infetado(a) com o COVID-19.	□	□	□	□	□

## Appendix B

Coronavirus Anxiety Scale

### Appendix B.1. English Version

How often have you experienced the following activities over the last 2 weeks?

**Table A3:** Items of the Coronavirus Anxiety Scale (English version).

Answers	Not at All	Rare, Less Than a Day or Two	Several Days	More Than 7 Days	Nearly Every Day over the Last 2 Weeks
1. I felt dizzy, lightheaded, or faint, when I read or listened to news about the coronavirus.	□	□	□	□	□
2. I had trouble falling or staying asleep because I was thinking about the coronavirus.	□	□	□	□	□
3. I felt paralyzed or frozen when I thought about or was exposed to information about the coronavirus.	□	□	□	□	□
4. I lost interest in eating when I thought about or was exposed to information about the coronavirus.	□	□	□	□	□
5. I felt nauseous or had stomach problems when I thought about or was exposed to information about the coronavirus.	□	□	□	□	□

### Appendix B.2. Portuguese Version

Tendo passado mais de uma hora (no total) por semana a pensar no coronavirus 19 e a ver notícias nas redes sociais e na televisão sobre ele, por favor indique em quantos dias teve os seguintes sintomas:

**Table A4:** Items of the Coronavirus Anxiety Scale (Portuguese version).

Respostas	Nunca	Raramente, Menos de um Dia ou Dois	Vários Dias	Mais do que 7 Dias	Quase Todos os Dias nas Últimas duas Semanas
1. Eu sentia-me tonto ou a desmaiar quando lia ou ouvia notícias sobre a COVID-19.	□	□	□	□	□
2. Tive problemas em adormecer ou manter o sono porque estava a pensar na COVID-19.	□	□	□	□	□
3. Eu senti-me paralisado(a) ou gelado(a) quando pensei ou fui exposto(a) a informações sobre a COVID-19.	□	□	□	□	□
4. Perdi o interesse em comer quando pensei ou fui exposto a informações sobre a COVID-19.	□	□	□	□	□
5. Senti náuseas ou problemas de estômago quando pensei ou fui exposto a informações sobre a COVID-19.	□	□	□	□	□
